# Circulating microRNAs as Biomarkers for Detection of Autologous Blood Transfusion

**DOI:** 10.1371/journal.pone.0066309

**Published:** 2013-06-20

**Authors:** Nicolas Leuenberger, Yorck Olaf Schumacher, Sylvain Pradervand, Thomas Sander, Martial Saugy, Torben Pottgiesser

**Affiliations:** 1 Swiss Laboratory for Doping Analyses, University Center of Legal Medicine, Geneva and Lausanne, Centre Hospitalier Universitaire Vaudois and University of Lausanne, Lausanne, Switzerland; 2 Department of Sports Medicine, University Hospital of Freiburg, Freiburg, Germany; 3 Aspetar Orthopedic and Sports Medicine Hospital, Doha, Qatar; 4 Genomic Technologies Facility, University of Lausanne, Lausanne, Switzerland; French National Center for Scientific Research - Institut de biologie moléculaire et cellulaire, France

## Abstract

MicroRNAs (miRNAs) are small non-coding RNAs that regulate various biological processes. Cell-free miRNAs measured in blood plasma have emerged as specific and sensitive markers of physiological processes and disease. In this study, we investigated whether circulating miRNAs can serve as biomarkers for the detection of autologous blood transfusion, a major doping technique that is still undetectable. Plasma miRNA levels were analyzed using high-throughput quantitative real-time PCR. Plasma samples were obtained before and at several time points after autologous blood transfusion (blood bag storage time 42 days) in 10 healthy subjects and 10 controls without transfusion. Other serum markers of erythropoiesis were determined in the same samples. Our results revealed a distinct change in the pattern of circulating miRNAs. Ten miRNAs were upregulated in transfusion samples compared with control samples. Among these, miR-30b, miR-30c, and miR-26b increased significantly and showed a 3.9-, 4.0-, and 3.0-fold change, respectively. The origin of these miRNAs was related to pulmonary and liver tissues. Erythropoietin (EPO) concentration decreased after blood reinfusion. A combination of miRNAs and EPO measurement in a mathematical model enhanced the efficiency of autologous transfusion detection through miRNA analysis. Therefore, our results lay the foundation for the development of miRNAs as novel blood-based biomarkers to detect autologous transfusion.

## Introduction

Autologous blood transfusions are prohibited in sports according to the World Anti-Doping Agency (WADA) [1]. There is, at present, no direct detection method, and the technique remains a significant problem in all endurance sport disciplines. A method that has provided acceptable sensitivity with low specificity is the detection of plasticizers and their metabolites, emanating during storage from the blood bags, which can be measured in urine [2]. Nevertheless, athletes might shift to use plasticizer-free blood bags to avoid this form of detection. The most promising approach to detect autologous transfusion is the Athlete Biological Passport (ABP), which is an indirect detection method, i.e., not the forbidden substance or method itself is detected, but rather its effects on certain biomarkers.

The ABP consists of longitudinal monitoring of different biomarkers to identify patterns suspicious of doping [3], [4]. The hematological module of the ABP uses blood markers to identify any modification of erythropoiesis [4]. In the same context, methods based on proteins as indirect markers for the detection of exogenous recombinant human erythropoietin (EPO) abuse have been described [5], [6]. In the future, new markers from proteomics, metabolomics, and genomics might therefore be integrated into the ABP to further enhance the detection of autologous blood transfusion.

MicroRNAs (miRNAs) are small regulatory, non-coding RNAs [7]. miRNAs primarily affect the stability of messenger RNA and/or the initiation and progression of protein synthesis, but broader regulatory roles have also been suggested [8], [9]. Recently, a number of miRNAs were found outside of cells. These “circulating miRNAs” are abundant in body fluids such as urine, saliva, and plasma [10], [11] and were linked to different specific pathophysiological conditions. Examples of such conditions include the association of plasma miR-122 with drug-induced liver injury [12], [13], and the identification of miR-144 as a promising biomarker to detect erythropoiesis-stimulating agents in anti-doping [14]. Compared to protein-based biomarkers, miRNA offers several advantages: miRNAs are very stable in various body fluids, expression of some miRNAs is restricted to specific tissues, and miRNA levels can be easily measured by common laboratory methods, including assorted signal amplification strategies [15].

Therefore, the purpose of this study was to investigate whether circulating miRNAs can be used as biomarkers to detect autologous blood transfusion in an anti-doping context. Other indirect parameters commonly used for the indirect detection of blood transfusion, hemoglobin concentration (Hb) and EPO concentration, were measured and compared with circulating miRNA data.

## Materials and Methods

### Subjects

**Figure 1:**
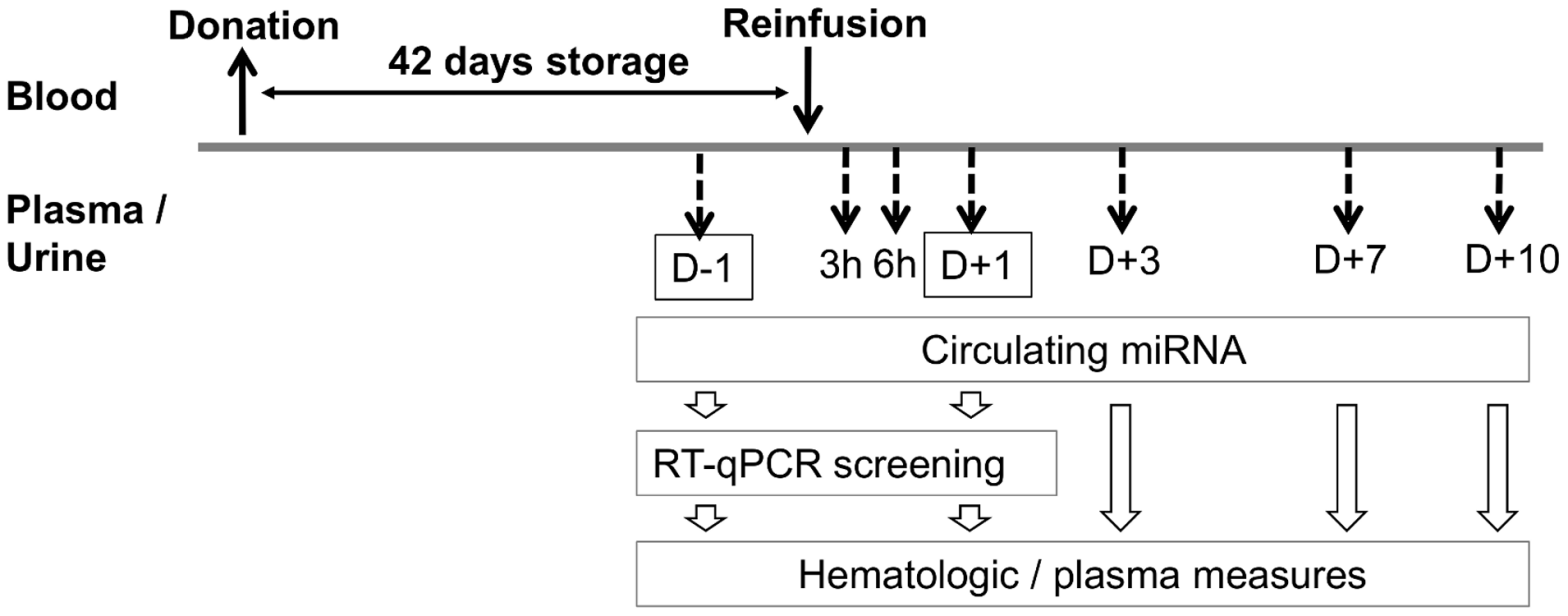
Study design. Samples were taken at different time points after blood reinfusion. For the control group, no samples were collected at 3 (3 h) and 6 hours (6 h).

In total, 20 healthy male subjects were recruited for the study. One group (Intervention, N = 10) underwent blood collection and autologous reinfusion 42 days later, and the remaining subjects served as a control group (Control, N = 10). The baseline characteristics of the study participants are outlined in Table 1. Plasma and urine samples were obtained the day before (D-1) and at several time points (3 h, 6 h (D0), D+1, D+3, D+7, and D+10) after autologous blood transfusion. Control samples were taken similarly, except with no samples collected at 3 h and 6 h on D0). The timeline of the study is illustrated in Figure 1.

**Table 1:** Baseline characteristics of groups.

Group	Intervention (n = 10)	Control (n = 10)
**Age, y (mean ± SD)**	25±2	25±2
**Weight, kg (mean ± SD)**	72±6.2	71.3±6.6
**Height, m (mean ± SD)**	179±5	180±5
**Hb, g/dL (mean± SD)**	15.7±5.0	15.2±1.2
	Hb : hemoglobin	

Anti-doping regulations were not violated since none of the subjects in the Intervention group was a license-holder in any sports discipline. The ethics committee at the University of Freiburg approved the study procedures (Amendment to Approval No. 177/06), and written informed consent was obtained from all subjects before participation.

### Blood Donation and Reinfusion

One full bag of blood (approximately 500 mL) was removed from each volunteer using international standard methods of transfusion medicine. The volume of packed red blood cells (RBCs) in the blood bag after processing and isolation was approximately 280 mL, with a hematocrit of 53–60%. During the preparation of packed RBCs, leukocytes were removed from whole blood by centrifugation and application of leukocyte filters. PAGGS-M was used as the storage solution. The RBCs were stored at 4°C in temperature-regulated refrigerators until reinfusion.

### Hematology and Clinical Chemistry

Venous blood samples from an antecubital vein were obtained following standard procedures. Blood was drawn into a 9-mL serum tube and a 2.7-mL ethylenediaminetetraacetate (EDTA) tube (Sarstedt S-Monovette, Sarstedt AG, Nürnbrecht, Germany). EDTA samples were analyzed for blood parameters using a fully automated hematology analyzer (Sysmex XE-2100, Sysmex, Norderstedt, Germany). Plasma from EDTA samples was centrifuged 10 min at 4000 rpm, collected, aliquoted, and frozen at −20°C until analysis. Serum samples were used for different analyses, which were performed in batch for all samples to reduce analytical variability. To test hemolysis in plasma, hemoglobin concentration was quantified using a plasma/low Hb photometer (HemoCue®, HemoCue AG, Wetzikon, Switzerland).

C-reactive protein (CRP), lactate dehydrogenase (LDH), EPO, and ferritin were measured in plasma using Immulite technology (Siemens AG, Erlangen, Germany) [16]. Human serum amyloid A (SAA) and surfactant protein D were measured in plasma using ELISA technology (Invitrogen, Carlsbad, USA and Biovendor, Brno, Czech Republic) in the Intervention group.

### MicroRNAs Quantitative Real-time PCR (qRT-PCR)

Total RNA, including miRNAs, was isolated from 200 µL plasma using the miRNAeasy kit (Qiagen) as described elsewhere [15], [14]. Total RNA samples of liver, heart, lungs, spleen, thymus, kidney, skeletal muscle, adipose tissue, and brain were derived from the FirstChoice® Human Total RNA Survey Panel (Invitrogen, Carlsbad, USA). Total RNA of whole blood was derived from a pool of 10 blood samples (plasma and cells).

For miRNA profiling, 4 µL of eluted RNA was used in a 20-µL reverse transcription (RT) reaction. UniSp6 Spike-in miRNA was added to the RT reaction mix. The resulting cDNA was diluted 110 times and profiled for the relative abundance of 369 miRNAs using miRNA Ready-to-Use PCR, Human panel I, V1.M quantitative real-time PCR (qRT-PCR) arrays (Exiqon), as described in the manufacturer’s instructions. PCR amplification was performed using a Roche LightCycler 480 real-time PCR system. Raw data analyses were performed with LightCycler 480 software (release 1.5.0). Following a procedure similar to that described by Pritchard et al. [17], the spike-in UniSp3 (represented in triplicate on the panel) and UniSp6 were used to normalize the array data. The average Cq (quantification cycle; standard name for Ct or Cp value) values of the 4 spike-ins (avgSpikes) were calculated for each array. The array with the lowest avgSpikes value was used as the baseline array. For the other arrays, the Cq values were shifted by the difference between the array avgSpikes and the avgSpikes of the baseline array. Cq values greater than or equal to 36 were considered beyond the limit of detection. For subsequent analyses, 242 miRNAs (of 382 total) with at least 4 Cq values (out of 18 samples) lower than 36 were retained. Differentially expressed miRNAs were identified using the R package LIMMA [17], using a linear model with the difference between D−1 and D+1 and the subject as factors. The false discovery rate (FDR) was computed using the Benjamini & Hochberg method [18]. The 95% confidence interval for the fold changes between the two time points was computed using the R function “t.test.”

For individual qPCR, RT and qPCR procedures were performed as described above. However, 20 µL of RT reaction was diluted 20× instead of 110×, and 4 µL of the diluted cDNA was used in 10-µL PCR amplification reactions. A non-template control was added to verify the specificity of the RT-qPCR. PCR efficiency and the dynamic range of quantification were similar for every tested sample. All sequences of primers are available on the Exiqon web site (http://www.exiqon.com/ls/_layouts).

To better understand the time course of changes in circulating miRNAs after blood reinfusion, individual qPCR assays on miR-26b, miR-30b, and miR-30c were performed. These miRNAs were selected due to their low Cq, high significance, and 3-fold change. Since miR-486 was not affected by blood reinfusion (Table 2), it was used as an endogenous internal control together with a spiked artificial miRNA (UniSP6). Normalization was performed using LightCycler 480 software (release 1.5.0) by annotating miR-486 and UniSp6 as “reference.”

**Table 2:** Most affected miRNAs by blood re-infusion.

miRNAs	Fold-Change(FC)	FC.low	FC.high	Average Cq	P.Value	FDR
**hsa-let-7d**	3.16	1.48	6.76	30.01	0.0028	0.105
**hsa-miR-26b**	3.03	1.42	6.48	28.67	0.0036	0.105
**hsa-miR-30c**	3.01	1.76	5.16	29.55	0.0005	0.105
**hsa-miR-30b**	2.98	1.48	6.00	27.42	0.0025	0.105
**hsa-miR-142-3p**	2.83	1.38	5.79	25.96	0.0039	0.105
**hsa-miR-103**	2.62	1.47	4.67	25.56	0.0021	0.105
**hsa-let-7g**	2.49	1.36	4.56	27.19	0.0039	0.105
**hsa-miR-26a**	2.40	1.33	4.34	27.46	0.0043	0.105
**hsa-let-7b**	2.15	1.33	3.48	27.75	0.0040	0.105
**hsa-miR-339-5p**	2.14	1.37	3.34	31.39	0.0030	0.105
**hsa-miR-486-5p**	1.01	−1.59	1.62	27.84	0.9623	0.994
**UniSp6**	1.02	−1.07	1.10	18.18	0.9076	0.956

### Combining Analyses

The formula (miR-30b/miR-Ctls)*(1/EPO) was used to test combination of analyses. Quantified cycles (Cq) from miR-30b were divided by miR-486-5p and UniSp6 Cq with LightCycler 480 software, release 1.5.0. The circulating miR-30b value was chosen due to its high concentration in plasma, fold change, and specificity. Because of the decrease of EPO concentration after autologous transfusion, the value 1/EPO was chosen to amplify miR-30b/miR-Ctls changes.

### Statistics

Statistical analysis was performed using two-tailed Student’s t-test. Quantitative data are expressed as mean ± SEM versus D-1, unless otherwise specified. *P* values less than 0.05 were considered to indicate statistical significance. Statistical analyses were performed using Stata software (StataIC 12, StataCorp). ANOVA was performed in R (function aov) using the sample collection time and subjects as factors. Post-hoc pairwise comparisons were performed with the R function TukeyHSD (Tukey’s Honestly Significant Differences). Comparisons with a *P* value <0.05 after adjustment for multiple comparisons were considered significant.

## Results

Screening of circulating miRNAs as autologous transfusion biomarkers was performed with qRT-PCR 1 day before and at various times after reinfusion (see Figure 1, Table S1). Data were analyzed using a linear model allowing pairing of the values by subjects (Table S2). The most significant miRNAs (FDR = 0.105) were selected for further investigations (Table 2). All these miRNAs demonstrated a fold-change increase larger than 2, and members of the same family were present, such as miR-let-7, miR-30, and miR-26 miRNAs. By contrast, no significant decrease of circulating miRNAs was observed after blood reinfusion (Table S2). The highest significant increases observed in plasma after blood reinfusion was in miR-let-7d, miR-26b, miR-30b, and miR-30c, with an average of 3-fold increase.

As the changes in miRNA levels could be due to different physiological responses related to autologous transfusion, two tissues with the highest expression of miRNAs were selected. In 9 of the 10 selected miRNAs (90%), the lungs were the tissue with most miRNA affected by autologous transfusion (Table 3). Additionally, an increase of surfactant protein D, a lung-specific protein, was observed (Figure S1).

**Table 3:** miRNAs among 10 tested organs.

**miRNAs**	Predominately expressed tissues
**miR-let-7d**	Heart, Lung
**miR-26b**	Lung, Spleen
**miR-30c**	Heart, Lung
**miR-30b**	Heart, Lung
**miR-142-3p**	Thymus,Spleen
**miR-103**	Brain, Lung
**miR-let-7g**	Lung, Heart
**miR-26a**	Lung, Skeletal muscle
**miR-let-7b**	Lung, brain
**miR-339-5p**	Kidney, Lung
**miR-486-5p**	Whole blood, Skeletal muscle

**Figure 2:**
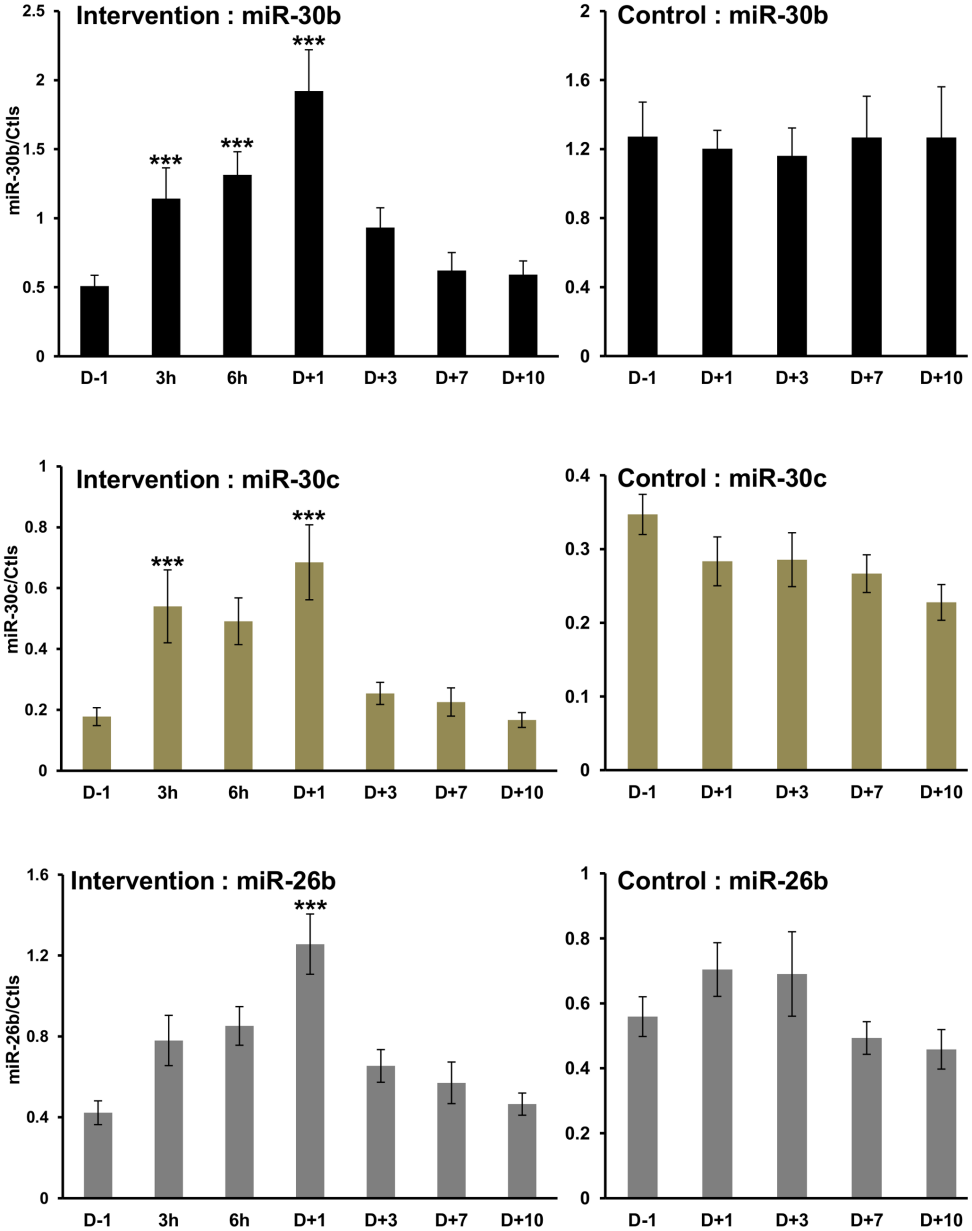
Circulating miRNA levels after autologous blood in the Intervention and Control groups. Levels of miR-30b, miR-30c and miR-26b in plasma samples collected from healthy volunteers after blood reinfusion (Intervention) or in the control group. The collection time points are indicated on the x-axis. Data were normalized to endogenous miR-486-5p and spike-in UniSp6 controls (Ctls). The values of miRNAs are the average of 10 independent samples from each time point. ***P≤0.001, statistical analyses are presented in Table 4.

Regarding the timeline, the reinfusion induced miR-30b, miR-30c, and miR-26b with a peak of induction one day (D+1) after blood transfusion (Figure 2, Intervention). ANOVA analyses demonstrated that the induction of miR-30b, miR-30c, and miR-26 was highly statistically significant (Table 4). Circulating miR-30b and miR-30c were already induced significantly at 3 hours after reinfusion. The control group showed no significant changes in any of the measured miRNAs (Figure 2, Control and Table 4).

**Table 4:** ANOVA analyses of individual qPCR.

Group	miRNA	F value	P value	Significant post-hoc pairwise comparisons				
**Intervention**	**26b**	6.642752	2.80E-05	D1-D-1, D10-D1, D3-D1, D7-D1				
**Intervention**	**30b**	7.946132	3.93E-06	D1-D-1, D10-3h, D10-6h, D10-D1, D3-D1, D7-D1				
**Intervention**	**30c**	5.594842	0.000149	D1-D-1, D10-3h, D10-D1, D3-D1, D7-D1				
**Control**	**26b**	1.077540	0.381995					
**Control**	**30b**	0.060522	0.992925					
**Control**	**30c**	1.248051	0.308249					

**Figure 3:**
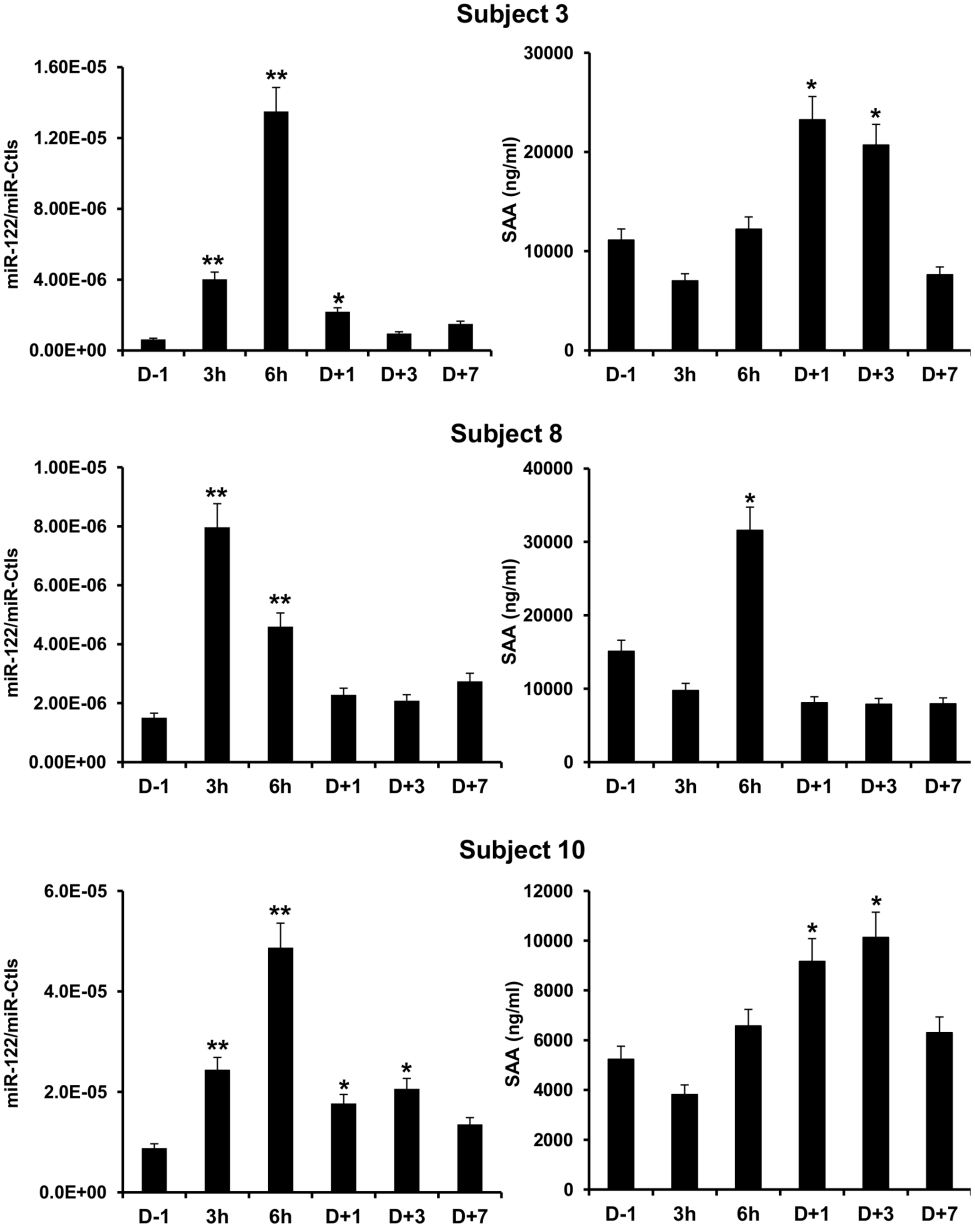
Liver-specific circulating miRNA miR-122 and level of serum amyloid A (SAA) after transfusion in three volunteers. The collection time points are indicated on the x-axis. Data were normalized to endogenous miR-486-5p and spike-in UniSp6 controls (Ctls). The relative changes of different miRNA levels are compared with D-1. Values represent the mean of three independent experiments ± SD. *P≤0.05 and **P≤0.01 versus D-1.

Three of 10 Intervention subjects displayed an increase of the liver-specific miR-122 [19] (Figure 3). Interestingly, SAA [20], a hepatic acute-phase reactant protein, was also elevated in the same subjects after reinfusion (Figure 3). No significant change was observed in the biochemical markers for leukocytes, CRP and LDH, after blood cell transfusion (Table 5).

**Table 5:** Hematologic variables before and during 10 days after transfusion.

Intervention	D–1	D+1	D+3	D+7	D+10
**Reti (%)**	0.75±0.03	0.80±0.04	0.80±0.03	0.72±0.04	0.72±0.04
**LDH (UI/L)**	158.7±3.6	170.7±5.7	167.4±2.8	183.5±7.0	171.4±6.8
**Leuk (10^3^/µL)**	5.3±0.2	6.1±0.5	5.6±0.2	6.4±0.3	6.2±0.3
**CRP (mg/dL)**	0.8±0.3	0.5±0.1	0.3±0.1	0.5±0.2	0.3±0.1
**Ery (10^6^/µL)**	5.08±0.05	5.32±0.06*	5.21±0.07	5.22±0.06	5.21±0.06
**Hb (g/dL)**	15.24±0.15	16.02±0.14*	15.71±0.13*	15.75±0.13*	15.7±0.13*
**EPO (UI/L)**	13.7±0.85	10.07±0.80	9.31±0.44*	9.67±0.86*	8.01±0.56**
**Ferritin (ng/mL)**	54.09±7.04	103.57±13.04*	87.24±9.4**	75.64±7.92**	82.43±8.17**
**Control**	**D-1**	**D+1**	**D+3**	**D+7**	**D+10**
**Reti (%)**	0.85±0.05	0.84±0.05	0.93±0.05	0.9±0.05	0.91±0.04
**LDH (UI/L)**	172.7±5.2	158.5±6.0	147.5±3.6	167±9.8	154.7±6.4
**Leuk (10^3^/µL)**	7.4±0.4	6.7±0.5	5.9±0.3	6.9±0.4	6.6±0.4
**CRP (mg/dL)**	0.8±0.2	0.6±0.1	0.7±0.1	1.2±0.3	1.2±0.3
**Ery (10^6^/µL)**	4.96±0.05	4.88±0.04	4.9±0.05	4.85±0.05	4.82±0.06
**Hb (g/dL)**	15.22±0.23	15.01±0.14	14.95±0.22	14.87±0.24	14.66±0.24
**EPO (UI/L)**	11.35±1.57	13.94±2.22	12.41±1.77	11.6±2.09	14.32±2.25
**Ferritin (ng/mL)**	71.3±8.64	77.6±9.57	71.65±9.49	73.19±9.44	70.57±9.47

Abbreviations : percentage of reticulocytes (Reti), lactate deshydrogenase (LDH), leukocytes (Leuk), C-reactive protein (CRP), erythrocytes (Ery), hemoglobin (Hb), erythropoietin (EPO) * and ** = significant difference (p≤0.05 and p≤0.01, resp.) from D-1 (one day before blood re-infusion).

The effect of autologous blood transfusion on traditional blood parameters was then compared to the change of circulating miRNAs. Erythrocyte count and hemoglobin concentration were increased 1 day after blood reinfusion (Table 5). No significant change was observed in the control group (Table 5). No significant change was observed in reticulocyte percentage after blood reinfusion (Table 5). Transfusion was associated with a significant, long-term decrease in EPO up to 10 days after blood reinfusion and an increase in ferritin (Table 5). No significant change was observed in the control group for these variables (Table 5).

**Figure 4:**
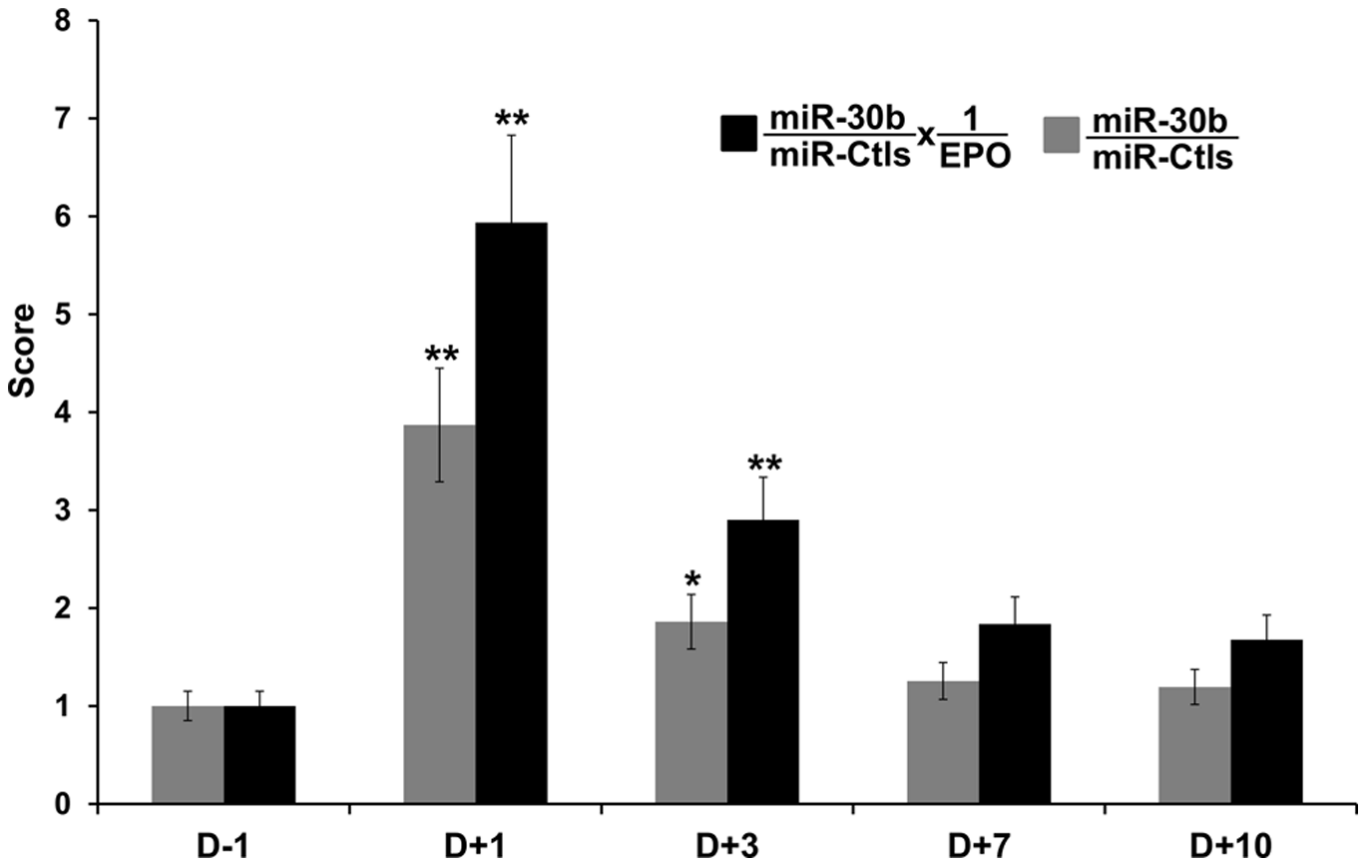
Combination of EPO and circulating miRNA. The levels of miR-30b and 1/EPO were compared with miR-30b alone. The collection time points are indicated on the x-axis. The relative changes of different miRNA levels are compared with D-1 (one day before transfusion). Data were normalized to endogenous miR-486-5p and spike-in UniSp6 controls (Ctls) The values for miRNAs are the average of 10 independent samples from each time point. Values are presented as mean ± SEM. *P≤0.05 and **P≤0.01 versus D-1.

Since circulating miRNA and EPO level in blood were both significantly influenced by autologous transfusion, the combination of the different variables was tested to increase the sensitivity of this biomarker approach. The amount of circulating miR-30b was divided by the EPO concentration. Three individual subjects are displayed as an example (Figure S2a, b and c). By dividing miRNA data by EPO concentration, the fold change compared to D-1 was increased by ∼50% compared to the values of circulating miR-30b alone. In addition, the statistical significance of the fold change 3 days after transfusion was increased, from *P* = 0.04 to *P* = 0.01 (Figure 4).

## Discussion

The key finding of our study is that the transfusion of autologous blood triggers an increase of selected circulating miRNAs in plasma compared to a non-transfused control group. These results suggest that circulating miRNAs may serve as biomarkers to detect autologous transfusion, especially when the change of miRNAs is combined with the long-term decrease of EPO after autologous transfusion in a mathematical model.

Screening for a potential “circulating miRNA signature” after blood reinfusion showed that 10 distinct miRNAs were candidates to serve as efficient biomarkers for the detection of autologous blood transfusion. The changes of miRNA levels could be due to different physiological responses to autologous transfusion. All plasma miRNAs have a tissue or cellular origin [21]. Thus, measurement of a specific circulating miRNA in different tissues is a strategy to confirm its origin [22].

Many circulating miRNAs found in plasma are highly expressed in various peripheral blood cells [17]. Thus, changes in miR-30b, miR-30c, and miR-26b after transfusion could be explained by an increase of cell lysis during storage leading to a higher level of plasma miRNAs after reinfusion. However, miRNAs such as miR-30b and miR-30c are mainly expressed in white blood cells [17]. As leukocytes were removed during the preparation of whole blood, the chance that miR-30b, miR-30c, and miR-26b are transfused with the packed RBC is limited.

Furthermore, as miR-486-5p is highly expressed in RBC [17] but does not increase after blood reinfusion, the release of miRNAs from destructed RBCs seems unlikely. In addition, our results were derived from whole blood samples at time points within hours and days after transfusion, when an adequate mixture of RBCs coming from the stored blood bag (280 mL packed RBCs, approximately 5–7% of total blood volume) and natural RBCs can be expected. Thus, even if hemolysis had occurred in the stored blood bag with subsequent reinfusion of released miRNAs, then their representation in whole blood would be low. In light of these arguments, it seems unlikely that hemolysis of stored blood is the main explanation for the increase of miR-30b, miR-30c, and miR-26b after blood reinfusion.

Tissue measurement demonstrated that most of the screened miRNAs were predominantly expressed in the lungs. Significant increase of surfactant protein D, a lung-specific blood protein [23], confirmed that pulmonary physiology is involved in the changes triggered by autologous blood transfusion (Figure S1). In studies with a murine transfusion model, prolonged storage of RBCs before transfusion into endotoxemic mice caused increases in lung chemokines [24]. In addition, so-called transfusion-related acute lung injury (TRALI) is a serious blood transfusion complication characterized by the acute onset of non-cardiogenic pulmonary edema following the transfusion of blood products [25]. Altogether, these observations suggest that autologous transfusion impacts pulmonary physiology, now mirrored by miRNA measurements.

The mechanism behind the secretion of miRNAs from the lungs into the bloodstream remains unclear. One proposed hypothesis is that all types of circulating miRNA in the biological fluids are merely byproducts of cellular activity [26], [27]. Indeed, autologous transfusion could trigger secretion of multiple lung-specific molecules such as proteins and miRNAs with poor selectivity. Our results concerning the secretion of human surfactant protein D and circulating miRNAs after blood reinfusion might support this “byproduct hypothesis.”

Reproducibility studies regarding miRNA analyses have emphasized the importance of choosing an endogenous control to counteract the physiological variance in miRNA levels between individuals. In our study, miR-486-5p and a spiked artificial UniSp6 miRNA were used as control miRNAs. No effect due to transfusion was observed with miR-486-5p (Table 1). Moreover, it was found at a high level in plasma (27.9 Ct) and had already proven suitable as an endogenous control in prior studies [28]. This miRNA was therefore chosen as an endogenous control to normalize the data.

The three best candidates selected for longitudinal assessment with individual normalized RT-qPCR over the study period were miR-30b, miR-26b, and miR-30c. These miRNAs demonstrated the highest plasma concentration, fold change, and statistical significance. In the same context, Let-7d was ruled out of the selection due to its low plasma concentration and specificity. Measurement over time revealed a peak increase at 1 day after blood reinfusion for miR-30b. A 4-fold change was observed for miR-30b and miR-30c, and a 3-fold change for miR-26b. The magnitude of the changes was unexpected considering the small physiological effect of autologous blood transfusion on peripheral blood markers. Moreover, the increase of miR-30b was still detectable after 3 days. These results demonstrated that a number of circulating miRNAs are influenced by reinfusion of stored blood.

Elevated hepatic miR-122 was correlated with the increase of SAA. Thus, genomic and protein analyses suggest that autologous transfusion could trigger a hepatic reaction in certain volunteers. In other studies, circulating miR-122 was demonstrated to increase after liver damage [12], [13]. Altogether, our data suggest that liver-secreted compounds may be interesting biomarkers to detect autologous blood transfusion when monitored longitudinally. In addition, the link between liver reaction and autologous blood transfusion could be useful in a clinical context.

Measurements of different blood parameters such as Hb were included in calculation models to detect autologous transfusion [3], [4], [29]. In our study, a significant increase of Hb was observed 1 day after blood reinfusion, and hemoglobin tended to remain elevated up to 10 days. These results are consistent with several previous reports [29], [30], [3], [4]. Interestingly, reticulocytes did not show any significant change, in contrast to previous reports [3], [4]. A similar result, however, was found in the study by Ashenden [29]. No significant change in general circulating inflammatory parameters such as leukocytes or CRP was observed in our study, as observed by others [30].

Our results demonstrated that autologous blood transfusion had a significant impact on EPO and ferritin concentration. Iron metabolism is impacted by stored blood in humans and mice [30], [31], [32]. In our study, the level of ferritin was elevated after blood transfusion and thus could be used as a potential biomarker to detect blood doping. Previously, serum ferritin was proposed as a biomarker to detect EPO abuse [6]. Supplemental oral iron intake is frequently used by athletes to prevent iron deficiency [33], [34] and such supplementation could easily interfere with the function of ferritin as a biomarker in the detection of doping, thereby reducing the specificity of this marker. The longitudinal measurement of EPO is a potentially effective method to unmask recombinant human EPO abuse in athletes [5], [6]. EPO is a key player in erythropoiesis. Therefore, the decrease of EPO concentration after the autologous transfusion in our study was expected.

Current use of the ABP emphasizes the advantages of using a combination of different biomarkers to detect doping in sports. From the perspective of our study, a combination of circulating miRNAs and EPO measurement might have greater discriminative power to detect autologous blood transfusion (Figure 4) than miRNAs alone. As both variables are measured in the same matrix, a single plasma-EDTA sample would be sufficient to perform the test. Such samples are already in use for the determination of hemoglobin and the other markers included in the ABP [4]; thus, no additional sample material would need to be taken from the athlete. Altogether, these data demonstrate that the combination of different biomarkers increases the efficiency of autologous blood transfusion detection. In this context, future studies should evaluate how a new marker consisting of miRNAs and EPO could be incorporated into the adaptive model of the ABP [35].

This study has several limitations. The influence of various confounding parameters such as high-altitude training, physical exercise, and the analytical robustness of the proposed biomarkers remains to be explored. However, our control group showed no significant inter-day variability for EPO or miR-30b. Nevertheless, long-term longitudinal measurements of the proposed miRNAs in elite athletes and controls are needed before our results can be implemented in a real anti-doping context or the adaptive model of the ABP.

In summary, our results revealed a distinct change in the pattern of circulating miRNAs, a specific “signature” after autologous blood transfusion. We observed that the origin of these miRNAs was related to pulmonary and liver tissues. The detection window of the observed changes was up to 3 days after blood transfusion. In the same context, the long-term changes observed in EPO might be combined with the aforementioned markers in a mathematical model to enhance the efficiency of detecting autologous blood transfusion. Therefore, our results may lay the foundation for the development of a combination of hematologic and genomic biomarkers as a novel blood-based strategy to detect autologous blood transfusion, perhaps as part of the adaptive model of the ABP.

## Supporting Information

Figure S1 **Levels of surfactant protein D after autologous blood transfusion.** Level of surfactant protein D in plasma samples collected from healthy volunteers after autologous blood transfusion. The collection time points are indicated on x-axis. The relative change of different miRNA levels are compared with D-1. The values of miRNAs are the average of 10 independent samples from each time points, values are presented as mean ± SEM. *P≤0.05 versus D-1.
(TIFF)

Figure S2 **Individual measurement of miR-30b, EPO and combined values.** Three subjects (A, B, C) were measured individually for normalized miR-30b, EPO concentration and the combination of both entities.
(PDF)

Table S1 **Cq of miRNAs at D-1 and D+1 for each subject in intervention group.**
(XLSX)

Table S2 **Total affected miRNAs by blood re-infusion.**
(XLSX)

## References

[1] Thevis M, Kuuranne T, Geyer H, Schanzer W (2012) Annual banned-substance review: analytical approaches in human sports drug testing. Drug Test Anal 4: 2–16. 22287289 10.1002/dta.415

[2] Monfort N, Ventura R, Platen P, Hinrichs T, Brixius K, et al (2011) Plasticizers excreted in urine: indication of autologous blood transfusion in sports. Transfusion 52: 647–657. 21895677 10.1111/j.1537-2995.2011.03331.x

[3] Pottgiesser T, Sottas PE, Echteler T, Robinson N, Umhau M, et al (2011) Detection of autologous blood doping with adaptively evaluated biomarkers of doping: a longitudinal blinded study. Transfusion 51: 1707–1715. 21382045 10.1111/j.1537-2995.2011.03076.x

[4] Schumacher YO, Saugy M, Pottgiesser T, Robinson N (2012) Detection of EPO doping and blood doping: the haematological module of the Athlete Biological Passport. Drug Test Anal 4: 846–853. 22374784 10.1002/dta.406

[5] Audran M, Gareau R, Matecki S, Durand F, Chenard C, et al (1999) Effects of erythropoietin administration in training athletes and possible indirect detection in doping control. Med Sci Sports Exerc 31: 639–645. 10331881 10.1097/00005768-199905000-00003

[6] Parisotto R, Gore CJ, Emslie KR, Ashenden MJ, Brugnara C, et al (2000) A novel method utilising markers of altered erythropoiesis for the detection of recombinant human erythropoietin abuse in athletes. Haematologica 85: 564–572. 10870111

[7] Ruvkun G (2008) The perfect storm of tiny RNAs. Nat Med 14: 1041–1045. 18841145 10.1038/nm1008-1041

[8] Holley CL, Topkara VK (2011) An Introduction to Small Non-coding RNAs: miRNA and snoRNA. Cardiovasc Drugs Ther 25: 151–159. 21573765 10.1007/s10557-011-6290-z

[9] Vasudevan S, Tong Y, Steitz JA (2007) Switching from repression to activation: microRNAs can up-regulate translation. Science 318: 1931–1934. 18048652 10.1126/science.1149460

[10] Mitchell PS, Parkin RK, Kroh EM, Fritz BR, Wyman SK, et al (2008) Circulating microRNAs as stable blood-based markers for cancer detection. Proc Natl Acad Sci U S A 105: 10513–10518. 18663219 10.1073/pnas.0804549105 PMC2492472

[11] Weber JA, Baxter DH, Zhang S, Huang DY, Huang KH, et al (2010) The microRNA spectrum in 12 body fluids. Clin Chem 56: 1733–1741. 20847327 10.1373/clinchem.2010.147405 PMC4846276

[12] Starkey Lewis PJ, Dear J, Platt V, Simpson KJ, Craig DG, et al (2011) Circulating microRNAs as potential markers of human drug-induced liver injury. Hepatology 54: 1767–1776. 22045675 10.1002/hep.24538

[13] Zhang Y, Jia Y, Zheng R, Guo Y, Wang Y, et al (2010) Plasma microRNA-122 as a biomarker for viral-, alcohol-, and chemical-related hepatic diseases. Clin Chem 56: 1830–1838. 20930130 10.1373/clinchem.2010.147850

[14] Leuenberger N, Jan N, Pradervand S, Robinson N, Saugy M (2011) Circulating microRNAs as long-term biomarkers for the detection of erythropoiesis-stimulating agent abuse. Drug Test Anal 3: 771–776. 22113880 10.1002/dta.370

[15] Andreasen D, Fog JU, Biggs W, Salomon J, Dahslveen IK, et al (2010) Improved microRNA quantification in total RNA from clinical samples. Methods 50: S6–9. 20215018 10.1016/j.ymeth.2010.01.006

[16] Mossuz P, Girodon F, Hermouet S, Dobo I, Lippert E, et al (2005) Serum erythropoietin measured by chemiluminescent immunometric assay: an accurate diagnostic test for absolute erythrocytosis. Clin Chem 51: 1018–1021. 15914784 10.1373/clinchem.2004.047365

[17] Pritchard CC, Kroh E, Wood B, Arroyo JD, Dougherty KJ, et al (2011) Blood cell origin of circulating microRNAs: a cautionary note for cancer biomarker studies. Cancer Prev Res (Phila) 5(3): 492–7. 22158052 10.1158/1940-6207.CAPR-11-0370 PMC4186243

[18] Benjamini Y, Hochberg Y (1995) Controlling the false discovery rate: a practical and powerful approach to multiple testing. J Roy Statist Soc Ser B (Methodological) 57: 289–300.

[19] Krutzfeldt J, Rajewsky N, Braich R, Rajeev KG, Tuschl T, et al (2005) Silencing of microRNAs in vivo with ‘antagomirs’. Nature 438: 685–689. 16258535 10.1038/nature04303

[20] Betts JC, Edbrooke MR, Thakker RV, Woo P (1991) The human acute-phase serum amyloid A gene family: structure, evolution and expression in hepatoma cells. Scand J Immunol 34: 471–482. 1656519 10.1111/j.1365-3083.1991.tb01570.x

[21] Duttagupta R, Jiang R, Gollub J, Getts RC, Jones KW (2011) Impact of cellular miRNAs on circulating miRNA biomarker signatures. PLoS One 6: e20769. 21698099 10.1371/journal.pone.0020769 PMC3117799

[22] Wang K, Zhang S, Marzolf B, Troisch P, Brightman A, et al (2009) Circulating microRNAs, potential biomarkers for drug-induced liver injury. Proc Natl Acad Sci U S A 106: 4402–4407. 19246379 10.1073/pnas.0813371106 PMC2657429

[23] Tzouvelekis A, Kouliatsis G, Anevlavis S, Bouros D (2005) Serum biomarkers in interstitial lung diseases. Respir Res 6: 78. 16042760 10.1186/1465-9921-6-78 PMC1215520

[24] Mangalmurti NS, Xiong Z, Hulver M, Ranganathan M, Liu XH, et al (2009) Loss of red cell chemokine scavenging promotes transfusion-related lung inflammation. Blood 113: 1158–1166. 19064726 10.1182/blood-2008-07-166264 PMC2635081

[25] Vlaar AP (2012) Transfusion-related acute lung injury: Current understanding and preventive strategies. Transfus Clin Biol 19: 117–124. 22682310 10.1016/j.tracli.2012.03.001

[26] Turchinovich A, Weiz L, Burwinkel B (2012) Extracellular miRNAs: the mystery of their origin and function. Trends Biochem Sci 37: 460–465. 22944280 10.1016/j.tibs.2012.08.003

[27] Turchinovich A, Weiz L, Langheinz A, Burwinkel B (2011) Characterization of extracellular circulating microRNA. Nucleic Acids Res 39: 7223–7233. 21609964 10.1093/nar/gkr254 PMC3167594

[28] Boeri M, Verri C, Conte D, Roz L, Modena P, et al (2011) MicroRNA signatures in tissues and plasma predict development and prognosis of computed tomography detected lung cancer. Proc Natl Acad Sci U S A 108: 3713–3718. 21300873 10.1073/pnas.1100048108 PMC3048155

[29] Ashenden M, Morkeberg J (2011) Net haemoglobin increase from reinfusion of refrigerated vs. frozen red blood cells after autologous blood transfusions. Vox Sang 101: 320–326. 21534982 10.1111/j.1423-0410.2011.01493.x

[30] Hod EA, Brittenham GM, Billote GB, Francis RO, Ginzburg YZ, et al (2011) Transfusion of human volunteers with older, stored red blood cells produces extravascular hemolysis and circulating non-transferrin-bound iron. Blood 118: 6675–6682. 22021369 10.1182/blood-2011-08-371849 PMC3242722

[31] Hod EA, Zhang N, Sokol SA, Wojczyk BS, Francis RO, et al (2010) Transfusion of red blood cells after prolonged storage produces harmful effects that are mediated by iron and inflammation. Blood 115: 4284–4292. 20299509 10.1182/blood-2009-10-245001 PMC2879099

[32] Kwiatkowski JL (2011) Management of transfusional iron overload - differential properties and efficacy of iron chelating agents. J Blood Med 2: 135–149. 22287873 10.2147/JBM.S13065 PMC3262345

[33] Dellavalle DM, Haas JD (2012) Iron status is associated with endurance performance and training in female rowers. Med Sci Sports Exerc 44: 1552–1559. 22382172 10.1249/MSS.0b013e3182517ceb

[34] McClung JP (2012) Iron status and the female athlete. J Trace Elem Med Biol 26: 124–126. 22572041 10.1016/j.jtemb.2012.03.006

[35] Sottas PE, Vernec A (2012) Current implementation and future of the Athlete Biological Passport. Bioanalysis 4: 1645–1652. 22831480 10.4155/bio.12.130

