# Supplementary figures and images for: Circulating microRNAs as Biomarkers for Detection of Autologous Blood Transfusion
Source: PLoS One. 2013 Jun 20;8(6):e66309. doi: 10.1371/journal.pone.0066309 (PMC3688786; doi:10.1371/journal.pone.0066309)

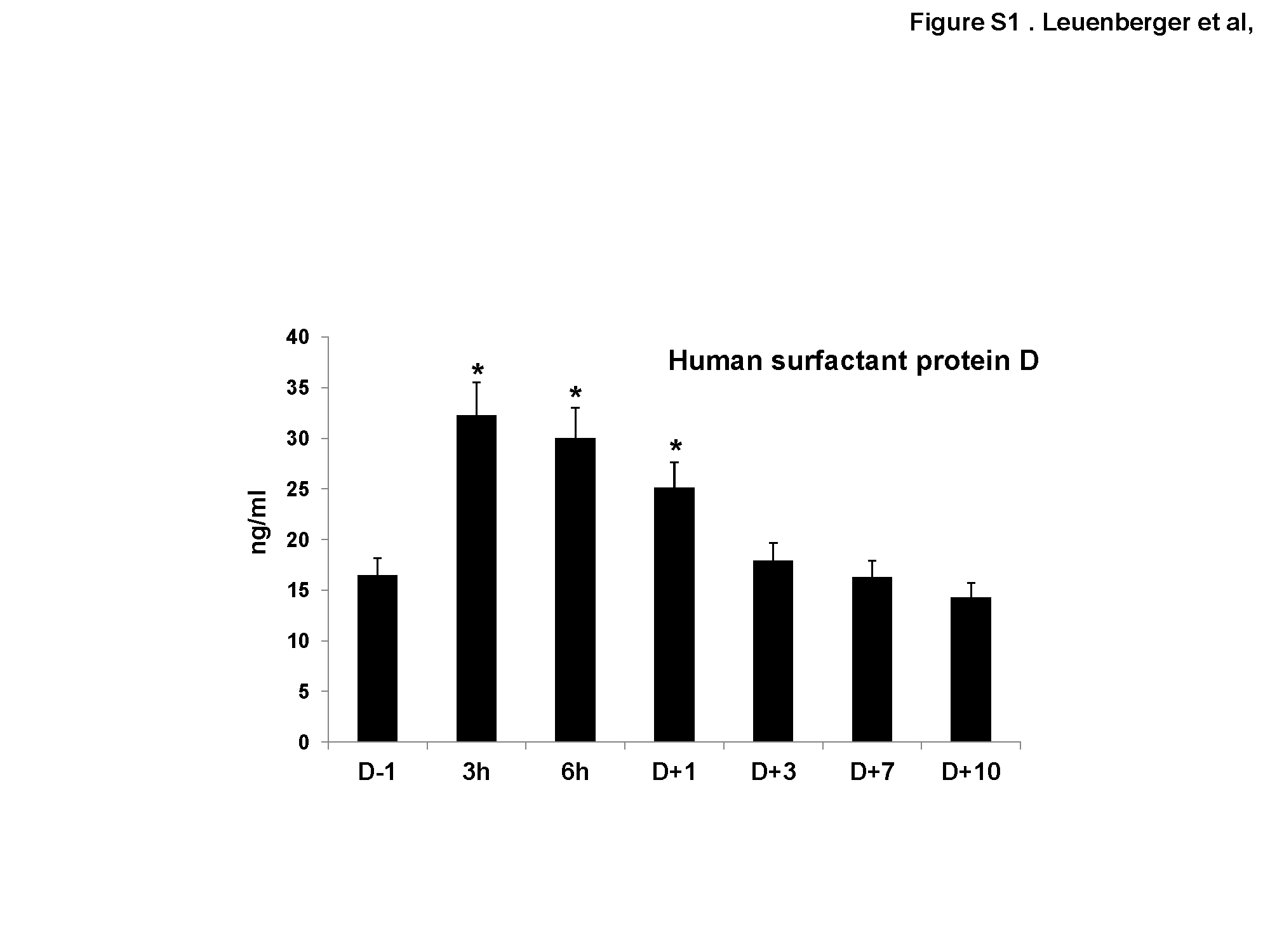

Supplement: Figure S1 — Levels of surfactant protein D after autologous blood transfusion. Level of surfactant protein D in plasma samples collected from healthy volunteers after autologous blood transfusion. The collection time points are indicated on x-axis. The relative change of different miRNA levels are compared with D-1. The values of miRNAs are the average of 10 independent samples from each time points, values are presented as mean ± SEM. *P≤0.05 versus D-1. (TIFF) [file pone.0066309.s001.tiff]

**A****Subject 5**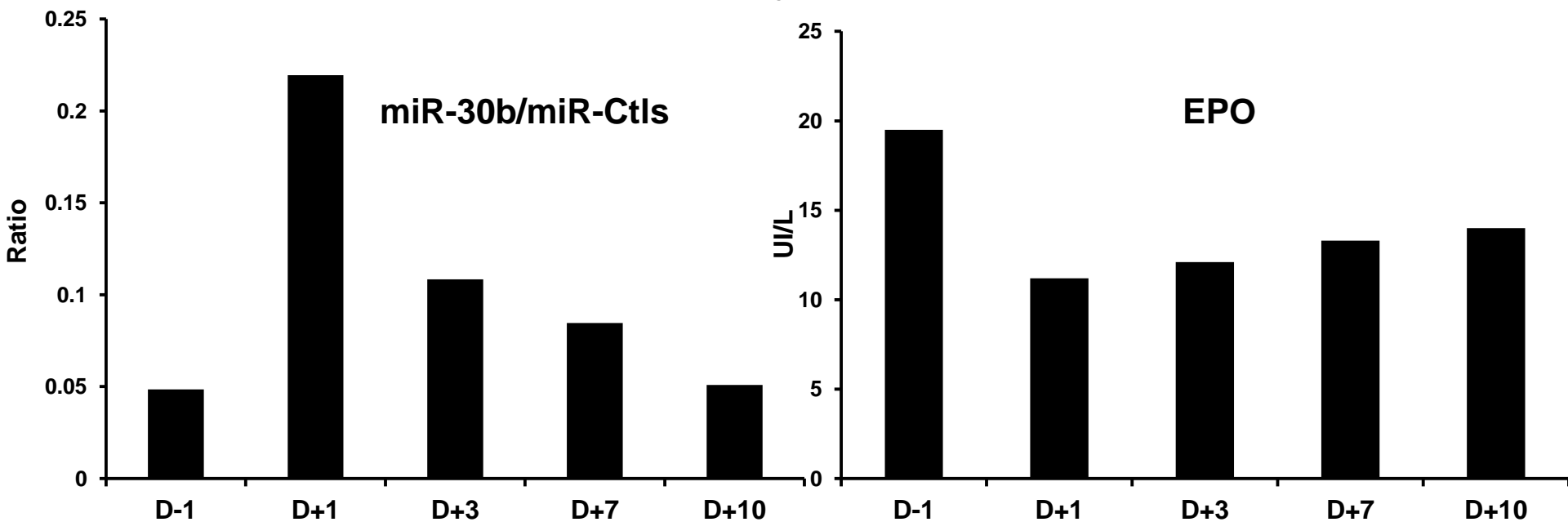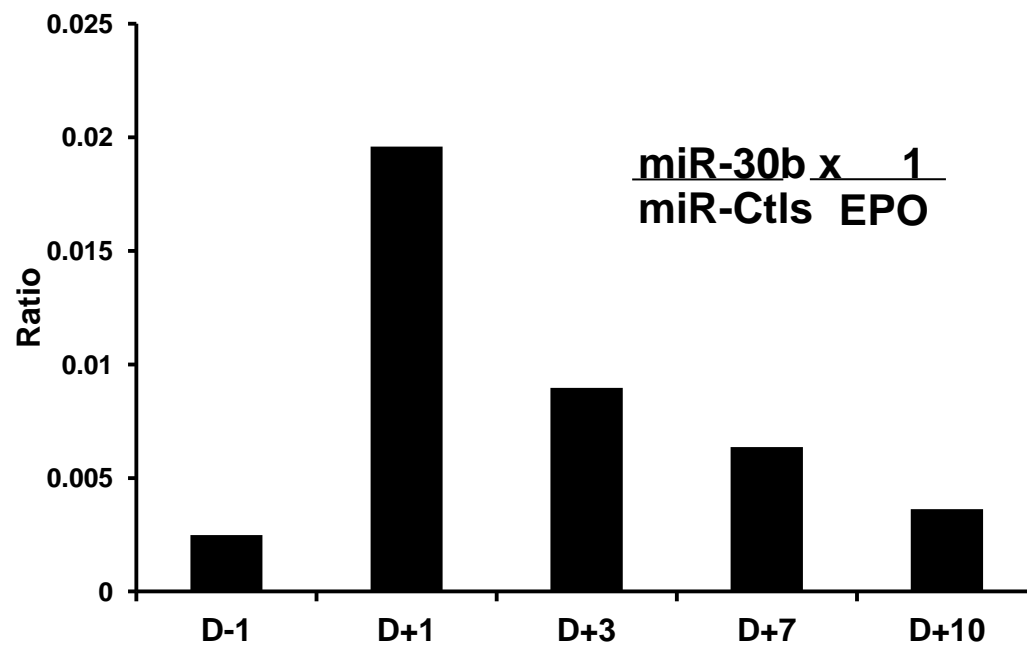

**B****Subject 7****miR-30b/miR-Ctls****EPO**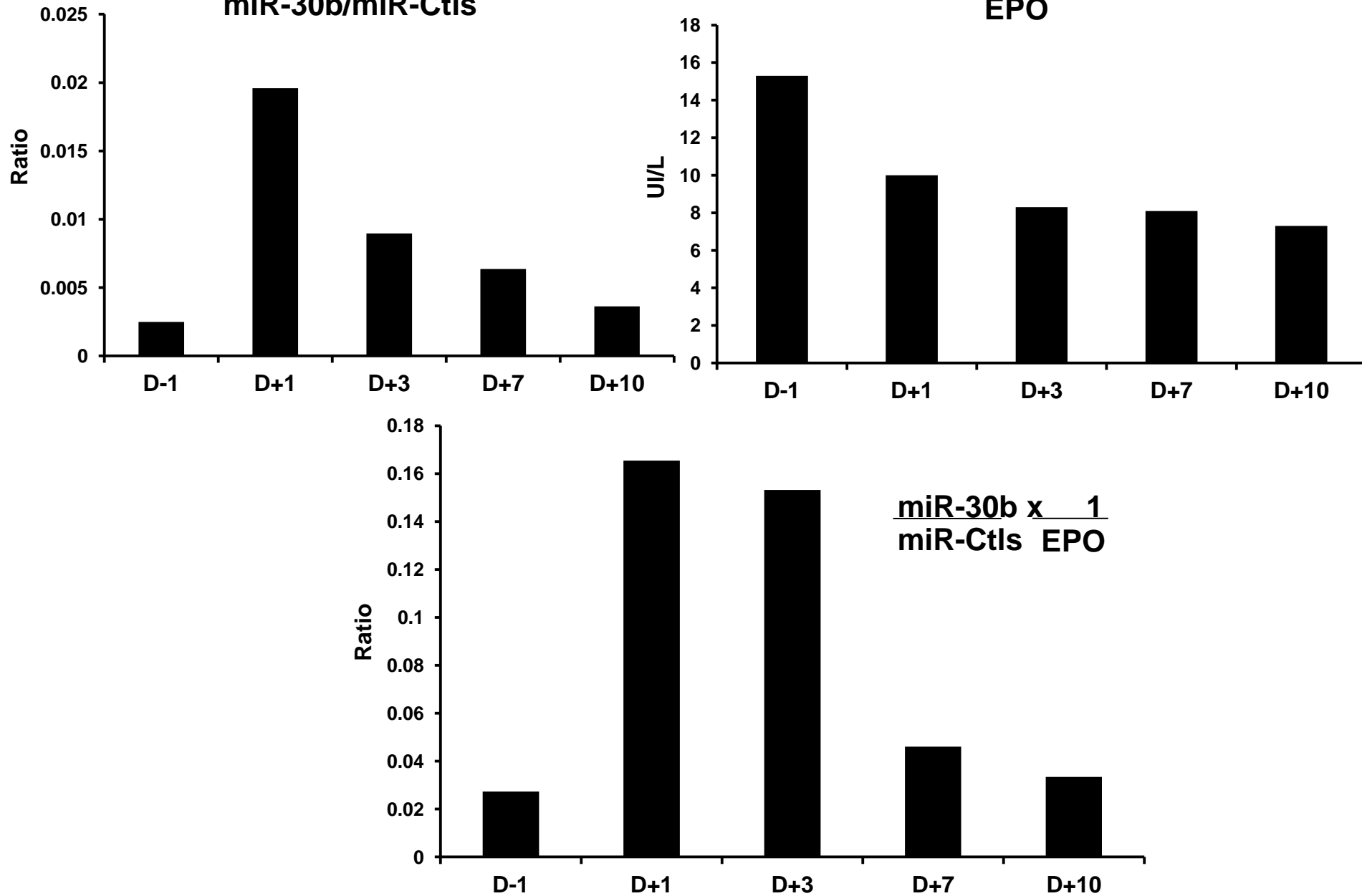

C

Subject 9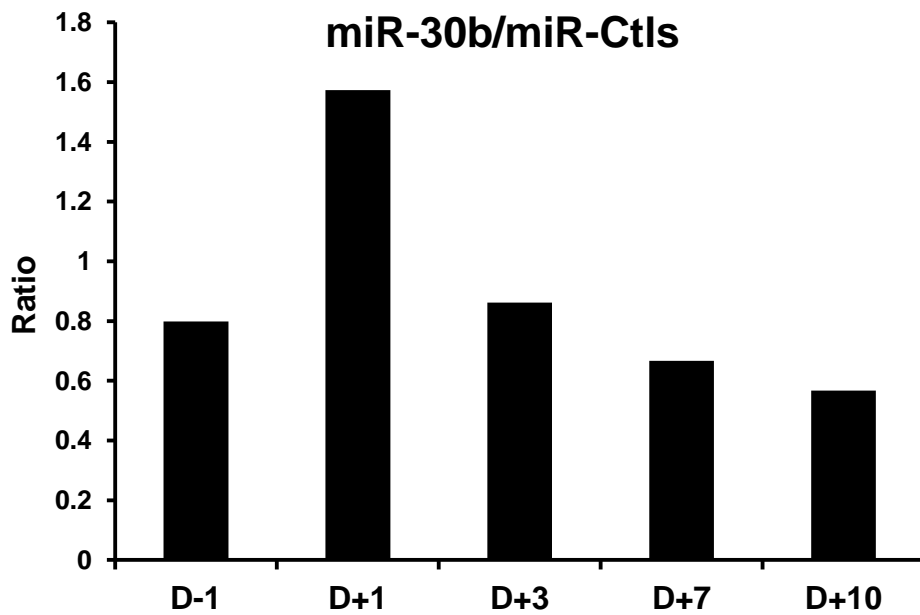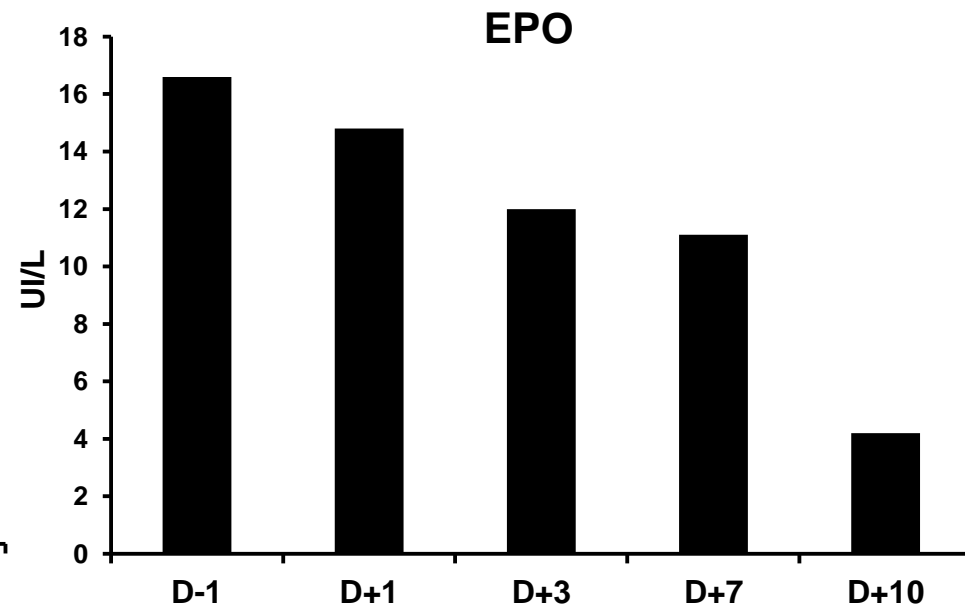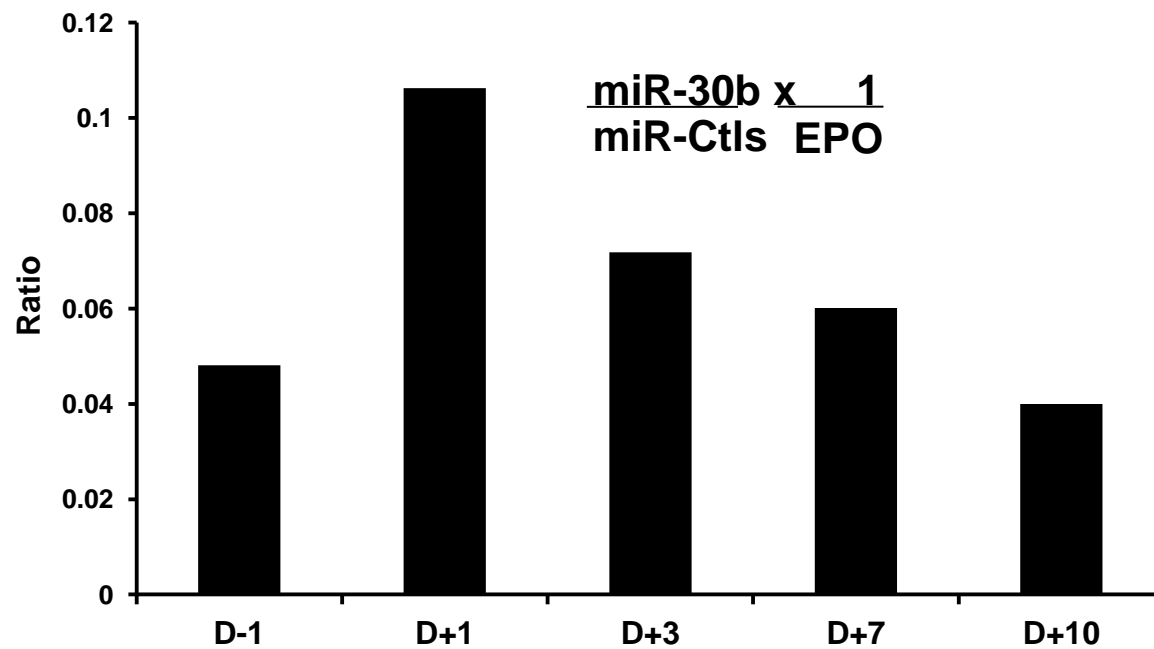

Supplement: Figure S2 — Individual measurement of miR-30b, EPO and combined values. Three subjects (A, B, C) were measured individually for normalized miR-30b, EPO concentration and the combination of both entities. (PDF) [file pone.0066309.s002.pdf]
